# Statutes of the University of Edinburgh, Relative to the Degree of M.D. 1833

**Published:** 1834-04-01

**Authors:** 


### STATUTES OF THE UNIVERSITY OF EDINBURGH, RELATIVE TO THE DEGREE OF M.D. 1833.

Sect. I. No one shall be admitted to the examinations for the degree of doctor of medicine, who has not been engaged in medical study for four years, during at least six months of each, either in the university of Edinburgh, or in some other university where the degree of M.D. is given; unless, in addition to three *anni medici* in an university, he has attended, during at least six winter months, the medical or surgical practice of a general hospital, which accommodates at least eighty patients, and during the same period a course of practical anatomy; in which case, three years of university study will be admitted.

Sect. II. No one shall be admitted to the examinations for the degree of doctor, who has not given sufficient evidence—

1. That he has studied, once at least, each of the following departments of medical science, under professors of medicine in this or in some other university, as already defined, viz.

During courses of six months—Anatomy, chemistry, materia medica and pharmacy, institutes of medicine, practice of medicine, surgery, midwifery, and the diseases peculiar to women and children, general pathology, practical anatomy (unless it has been attended in the year of extra-academical study allowed by Sect. I.)

During courses of six months, or two courses of three months—Clinical medicine: that is, the treatment of patients in a public hospital, under a professor of medicine, by whom lectures on the cases are given.

During courses of at least three months—Clinical surgery, medical jurisprudence, botany, natural history, including zoology.

2. That, in each year of his academical studies in medicine, he has attended at least two of the six months' courses of lectures above specified, or one of these and two of the three months' courses.

3. That, besides the course of clinical medicine already prescribed, he has attended, for at least six months of another year, the medical or surgical practice of a general hospital, either in Edinburgh or elsewhere, which accommodates not fewer than eighty patients.

4. That he has attended for at least six months, by apprenticeship or otherwise, the art of compounding and dispensing drugs at the laboratory of an hospital, dispensary, member of a surgical college or faculty, licentiate of the London or Dublin Society of Apothecaries, or a professional chemist and druggist.

5. That he has attended, for at least six months, by apprenticeship or otherwise, the out-practice of an hospital, or the practice of a dispensary, or that of a physician, surgeon, or member of the London or Dublin Society of Apothecaries.

Sect. III. No one shall obtain the degree of doctor who has not studied, in the manner already prescribed, for at least one year previous to his graduation, in the university of Edinburgh.

Sect. IV. Every candidate for the degree of medicine must deliver, before the 24th of March of the year in which he proposes to graduate, to the dean of the faculty of medicine:

1st. A declaration, in his own handwriting, that he is twenty-one years of age, or will be so before the day of graduation; and that he will not be then under articles of apprenticeship to any surgeon or other master.

2dly. A statement of his studies, as well in literature and philosophy as in medicine, accompanied with proper certificates.

3dly. A medical dissertation, composed by himself, in Latin or English; to be perused by a professor, and subject to his approval.

Sect. v. Before a candidate be examined in medicine, the medical faculty shall ascertain, by examination, that he possesses a competent knowledge of the Latin language.

Sect. vi. If the faculty be satisfied on this point, they shall proceed to examine him, either *viva voce* or in writing, first, on anatomy, chemistry, botany, institutes of medicine, and natural history, bearing chiefly on zoology; and secondly, on materia medica, pathology, practice of medicine, surgery, midwifery, and medical jurisprudence.

Sect. vii. Students who profess themselves ready to submit to an examination on the first division of these subjects, at the end of the third year of their studies, shall be admitted to it at that time.

Sect. viii. If any one, at these private examinations, be found unqualified for the degree, he must study for another year two of the subjects prescribed in section ii., under professors of medicine, in this or in some other university, as above defined, before he can be admitted to another examination.

Sect. ix. Should he be approved of, he will be allowed, but not required, to print his thesis; and, if printed, forty copies of it must be delivered before the 25th day of July to the dean of the medical faculty.

Sect. x. If the candidate have satisfied the medical faculty, the dean shall lay the proceedings before the Senatus Academicus, by whose authority the candidate shall be summoned, on the 31st of July, to defend his thesis; and finally, if the senate think fit, he shall be admitted, on the first lawful day of August, to the degree of doctor.

Sect. xi. The Senatus Academicus, on the day here appointed, shall assemble at ten o'clock A.M., for the purpose of conferring the degree; and no candidate, unless a sufficient reason be assigned, shall absent himself, on pain of being refused his degree for that year.

Sect. xii. Candidates for graduation shall be required to produce evidence of their having conformed to those regulations which were in force at the time they commenced their medical studies in a university.*

JAMES SYME,
**Prof. of Clinical Surgery, Dean of Faculty of Medicine.**

W. HAMILTON,
**Secretary to the Senatus Academicus.**

* Candidates who commenced their university studies before 1825 will be exempted from the fourth year of attendance (Sect. i), from the additional hospital attendance (Sect. ii. art. 3), from the necessity of a year's study in Edinburgh (Sect. iii.), and from any attendance on clinical surgery, medical jurisprudence, natural history, military surgery, practical anatomy, pathology, and surgery distinct from anatomy.

Those who commenced between 1825 and 1831 will be exempted from attendance on general pathology, and also on surgery distinct from anatomy.

Those who commenced between 1825 and 1833 will be required to attend only two of the following classes, viz. clinical surgery, medical jurisprudence, natural history, military surgery, practical anatomy.

And those who commenced before 1833 will be exempted from the attendance specified in Sect. ii., arts. 4 and 5.

N.B. The attendance on midwifery in an university (Sect. i. art. 1,) is required of all candidates.

